# Oxysterol Induces Expression of 60 kDa Chaperone Protein on Cell Surface of Microglia

**DOI:** 10.3390/ijms25169073

**Published:** 2024-08-21

**Authors:** Koanhoi Kim, Hyok-rae Cho, Bo-young Kim, Jaesung Kim, Dongha Park, Ryuk Jun Kwon, Yonghae Son

**Affiliations:** 1 Department of Pharmacology, School of Medicine, Pusan National University, Yangsan 50612, Republic of Korea; koanhoi@pusan.ac.kr (K.K.); rlawotjd1029@naver.com (J.K.); propertopic@pusan.ac.kr (D.P.); 2 Department of Neurosurgery, College of Medicine, Kosin University, Busan 49267, Republic of Korea; drchr@hanmail.net; 3 Research Institute for Convergence of Biomedical Science and Technology, Pusan National University Yangsan Hospital, Yangsan 50612, Republic of Korea; kimboyoung@pusan.ac.kr; 4 Family Medicine Clinic and Research Institute for Convergence of Biomedical Science and Technology, Pusan National University Yangsan Hospital, Yangsan 50612, Republic of Korea; 5 Department of Family Medicine, School of Medicine, Pusan National University, Yangsan 50612, Republic of Korea

**Keywords:** 25-hydroxycholesterol, 27-hydroxycholesterol, HSP60, microglia, neuroinflammation

## Abstract

Microglia, essential immune cells in the brain, play crucial roles in neuroinflammation by performing various functions such as neurogenesis, synaptic pruning, and pathogen defense. These cells are activated by inflammatory factors like β-amyloid (Aβ) and oxysterols, leading to morphological and functional changes, including the secretion of inflammatory cytokines and the upregulation of MHC class II molecules. This study focused on identifying specific markers for microglial activation, with a particular emphasis on the roles of oxysterols in this process. We used the HMC3 human microglial cell line to investigate the induction of heat shock protein 60 (HSP60), a chaperonin protein by oxysterols, specifically in the presence of 25-hydroxycholesterol (25OHChol) and 27-hydroxycholesterol (27OHChol). Our findings obtained by the proteomics approach revealed that these oxysterols significantly increased HSP60 expression on microglial cells. This induction was further confirmed using Western blot analysis and immunofluorescence microscopy. Additionally, Aβ_1–42_ also promoted HSP60 expression, indicating its role as a microglial activator. HSP60 involved in protein folding and immune modulation was identified as a potential marker for microglial activation. This study underscores the importance of HSP60 in the inflammatory response of microglia, suggesting its utility as a target for new therapeutic approaches in neuroinflammatory diseases such as Alzheimer’s disease (AD).

## 1. Introduction

Microglia are important immune cells that play crucial roles in the neuroinflammatory response of the brain. These cells are multitasking specialists performing several roles, including (1) cleaning the brain by removing accumulating dying neurons; (2) facilitating neurogenesis and axonal growth; (3) eliminating unwanted synaptic connections; (4) remodeling vessels by promoting angiogenesis and tip fusion; (5) protecting the brain against pathogens; and (6) removing cell debris and promoting wound repair [1,2]. Microglia are activated by exposure to inflammatory factors such as β-amyloid (Aβ), oxysterols, and pathogens [3,4,5]. Upon activation, these cells undergo changes in shape, number, and branching complexity [4]. Activated microglia secrete inflammatory cytokines at the site of inflammation, including IL-1β, and express increased levels of MHC class II (MHC II) molecules [6]. Some studies have shown that the activation of microglia amplifies neuroinflammation in Alzheimer’s disease (AD) and contributes to its pathology [3,7,8].

Oxysterols are generated by the enzymatic oxidation or autoxidation of cholesterol [9]. The most abundant oxysterol in a normal brain is 24s-hydroxycholesterol (24sOHChol), followed by 25-hydroxycholesterol (25OHChol) and 27-hydroxycholesterol (27OHChol). However, the levels of certain oxysterols, including 25OHChol, 27OHChol, 7-ketocholesterol (7K), 7α-hydroxycholesterol (7αOHChol), and 7β-hydroxycholesterol (7βOHChol), increase, while the levels of 24sOHChol decrease in the brains of AD patients [10,11]. Among them, 25OHChol and 27OHChol are involved in inflammatory immune responses through the activation of immune cells and the induction of inflammatory factors [12,13,14]. Additionally, a previous study reported that oxysterols induce microglial activation, such as the expression of IL-1β and MHC II, which are markers of microglia activation [15].

Identifying specific markers for microglial activation is important, as they can be utilized as targets for new drugs or as guidance for novel treatments. This study explores multiple approaches to discover the novel markers and investigates the relationship between microglia activation and neuroinflammatory diseases, thereby contributing to the development of new drugs and treatments.

## 2. Results

### 2.1. Induction of HSP60 by the Oxysterols on the Microglia

**Figure 1:**
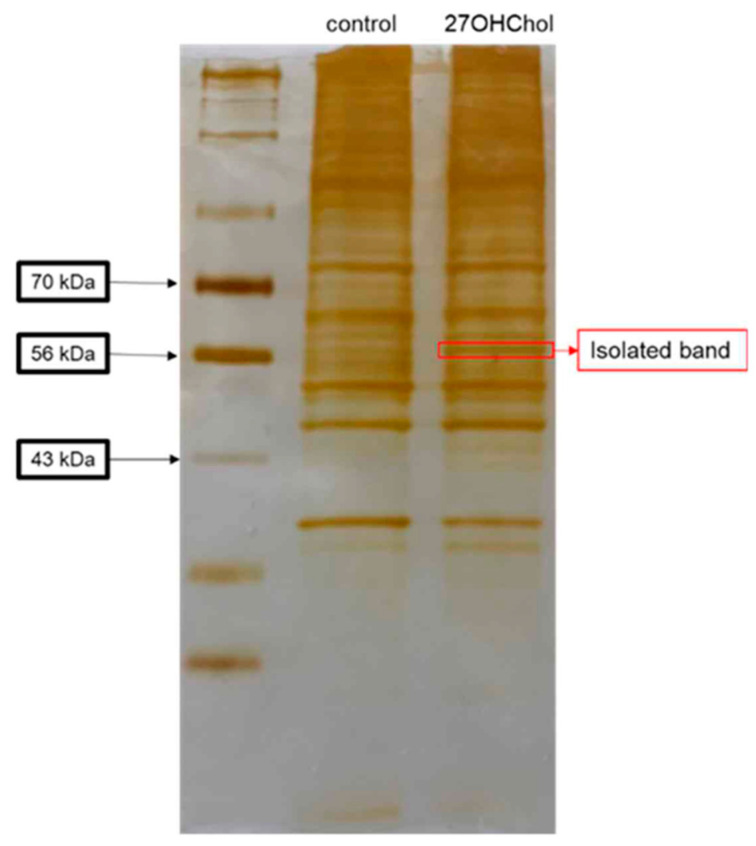
Isolated protein band from silver-stained gel. HMC3 cells (1 × 10^6^ cells) were stimulated with or without 1 μg/mL of 27OHChol (concentration of stock: 2 mg/mL) for 48 h, and then surface proteins were isolated with PIERCE^®^ Cell Surface Protein Isolation Kit. The proteins were separated in 10% SDS-contained polyacrylamide gel, and then the gel was stained with PIERCE^®^ Silver Stain Kit. A band was isolated from the gel (red square) and analyzed by MALDI-TOF-MS/MS.

To identify cell surface proteins whose expression increased on activated microglia, we investigated proteins induced after treatment with 27OHChol. The isolated cell surface proteins were visualized via silver staining (Figure 1), with an intensified band subjected to analysis through MALDI-TOF MS/MS. Proteomic analysis suggested the heightened presence of HSP60 (Supplementary Materials).

**Figure 2:**
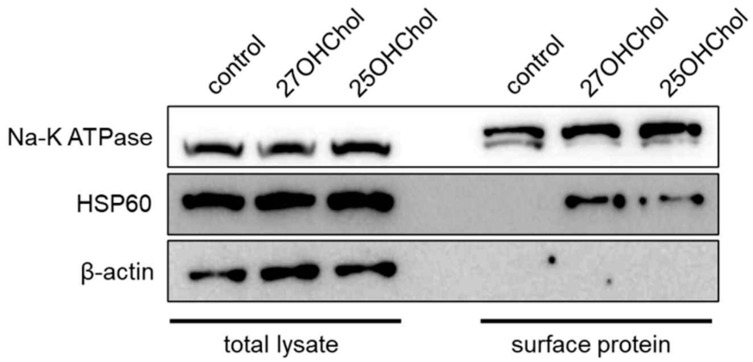
Expression of HSP60 on microglia treated with the oxysterols. HMC3 cells (1 × 10^6^ cells) were treated with 1 μg/mL of 25OHChol and 27OHChol (concentration of stocks: 2 mg/mL) for 48 h. Cell surface proteins harvested from the cells were analyzed with Western blotting. Na-K ATPase is a control for cell surface protein, and β-actin is a loading control. The presented data are representative of three independent experiments.

**Figure 3:**
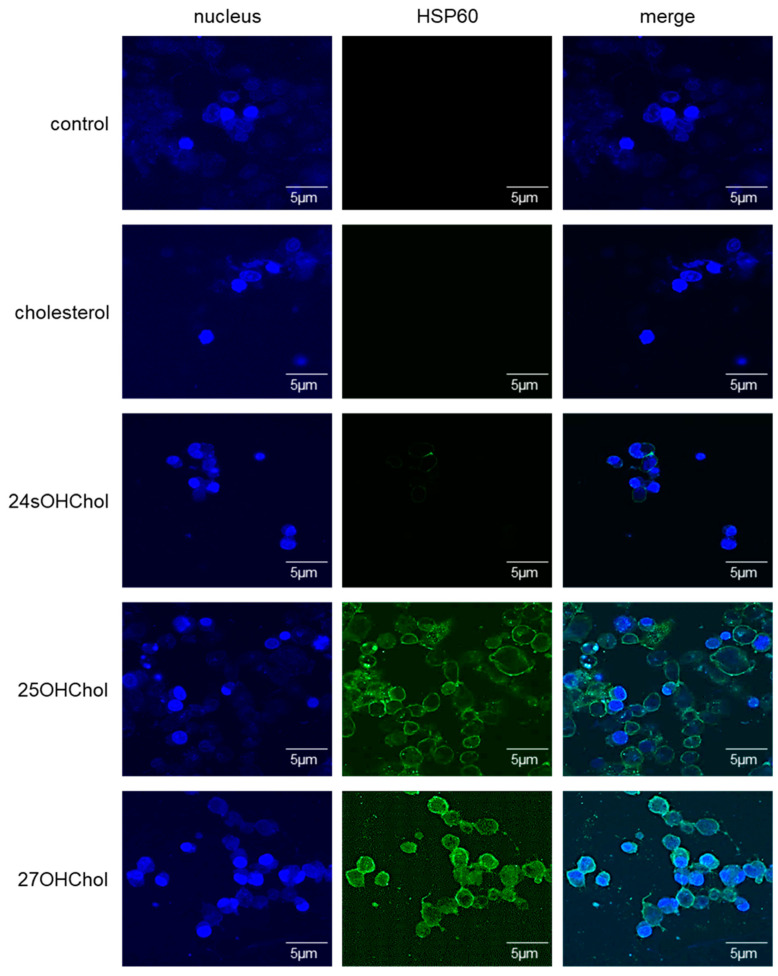
Visualization of the HSP60 expression on microglia treated with the oxysterols. HMC3 cells seeded on 0.2% gelatin-coated coverslip were stimulated with 1 μg/mL of cholesterol, 24sOHChol, 25OHChol, and 27OHChol (concentration of stocks: 2 mg/mL) for 48 h. The cells were incubated with antibody against HSP60, followed by fluorescence-conjugated 2nd antibody. The samples were visualized with confocal microscopy. The presented data are representative of three independent experiments.

To corroborate the proteomic findings, we examined whether HSP60 induction occurred upon treatment with 25OHChol and 27OHChol on microglial cells. The protein levels of HSP60 increased following oxysterol treatment (Figure 2). Furthermore, the expression of this chaperone protein on microglial cells was observed via fluorescence microscopy (Figure 3). Green signals indicative of HSP60 were discerned on the cell surfaces of microglia stimulated by 25OHChol and 27OHChol. Conversely, such signals were absent in cells incubated with control substances, cholesterol, and 24sOHChol. These findings collectively suggest that 25OHChol and 27OHChol promote the expression of HSP60 in microglia.

### 2.2. Roles of aβ in the Induction of HSP60 on Microglial Cells

**Figure 4:**
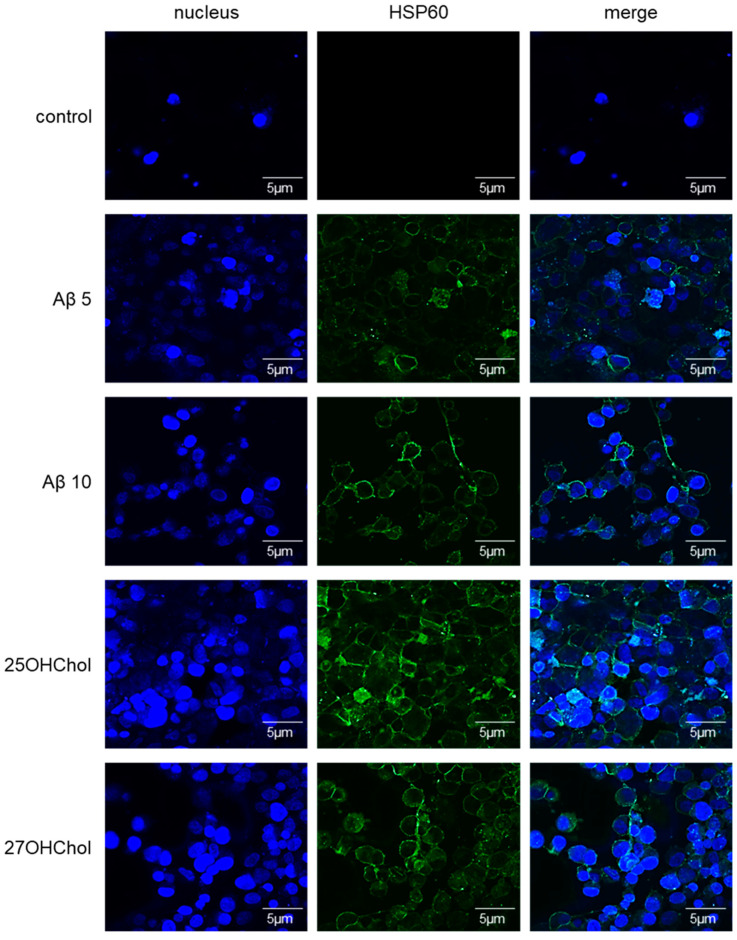
Visualization of the HSP60 expression on microglia treated with Aβ_1–42_. The cells seeded on 0.2% gelatin-coated coverslip were stimulated with 5 or 10 μM of Aβ_1–42_ and 1 μg/mL of 25-/27OHChol (concentration of stocks: 2 mg/mL) for 48 h. The cells were incubated with antibody against HSP60, followed by fluorescence-conjugated 2nd antibody. The samples were visualized with confocal microscopy. The presented data are representative of three independent experiments.

To ascertain whether Aβ influenced the expression of HSP60 on HMC3 cells, an investigation was conducted utilizing Aβ_1–42_. Cells were stimulated with Aβ_1–42_ along with 25OHChol and 27OHChol, followed by fluorescence labeling and visualization using a confocal microscope (Figure 4). The findings revealed that HSP60 was expressed on the cell surface of cells treated with Aβ_1–42_, mirroring the expression observed in cells treated with the oxysterols. These findings substantiate that Aβ, recognized as a microglial activator, also triggers the induction of the chaperone protein in microglial cells.

## 3. Discussion

Neuroinflammatory diseases such as AD, Parkinson’s disease (PD), and stroke have emerged as significant challenges in contemporary society. Numerous studies have investigated the factors underlying neuroinflammation. Notably, our recent paper elucidated that 25OHChol and 27OHChol are among the crucial neuroinflammatory factors [15]. In this study, oxysterols were found to induce microglial activation, evidenced by the upregulation of IL-1β and MHC II, which serve as indicators of microglial activation.

We identified HSP60, also known as chaperonin, which is expressed on microglial cells stimulated with oxysterols. Previous studies have demonstrated a correlation between HSP60 and microglial activation. Swaroop et al. observed a significant increase in IL-1β and HSP60 levels in non-infectious brain diseases such as glioma, AD, PD, and stroke, reporting that HSP60 regulates the expression of IL-1β in activated microglial cells [16]. Furthermore, this research group illustrated that HSP60 acts as a regulator of IL-1β-induced microglial inflammation [17]. These findings corroborate our results, demonstrating the upregulation of HSP60 expression induced by 25OHChol and 27OHChol in microglial cells.

The increase in HSP60 on the cell surface of microglial cells has multiple implications, necessitating an understanding of chaperonin. HSP60, a critical chaperonin protein in cellular stress responses, ensures proper protein folding and modulates immune reactions, aiding in cell survival and recovery [18,19]. Functionally, elevated HSP60 signifies activated microglia, promoting inflammatory responses via cytokine secretion (e.g., IL-1β), thus facilitating immune reactions to neural damage or pathogens [16,17]. HSP60 also protects cells under stress, preventing protein damage and aiding tissue recovery, which is crucial in conditions like AD, PD, and stroke where microglial overactivation and inflammation are pivotal [20].

Many specific markers for the activation of microglia have been identified, including ionized calcium-binding adapter molecule 1 (Iba1), CD11b (known as Mac-1), CD68 (a scavenger receptor), translocator protein (TSPO), MHC II, CD40, and triggering receptor expressed on myeloid cells 2 (TREM2) [21,22]. TREM2 also has been reported to exhibit increased expression in neurodegenerative diseases such as AD. Our study demonstrates that, in addition to these markers, HSP60 can also serve as a specific indicator of activated microglia.

A comparative analysis of microglial activation markers, such as Iba1, CD11b, and TREM2, alongside HSP60, is crucial for accurately understanding and diagnosing microglial activation states. Here, we provide a concise explanation of the functions of each marker and a comparative analysis with HSP60.

Iba1 is a well-established marker of microglial activation, with its efficacy validated in numerous studies. However, information regarding the specific functions or states of activated microglia remains limited. CD11b reflects the activation status of microglia involved in inflammatory responses, making it valuable for inflammation-related research. However, since CD11b is also expressed in other immune cells, such as macrophages, it may lack specificity as a microglial marker. TREM2’s association with the protective roles of microglia makes it a focal point in neurodegenerative disease research. Yet, changes in TREM2 expression are observed only under specific pathological conditions, making it challenging to generalize across all cases. HSP60, due to its specific role in cellular stress responses, can shed light on unique aspects of microglial activation, offering distinct information compared to more general markers like Iba1, CD11b, and TREM2. However, HSP60 is not universally expressed in all microglia but only under certain conditions, which may limit its utility as a general activation marker.

Given its close association with cellular stress responses in microglia, HSP60, when used alongside other markers, can provide a more detailed assessment of microglial activation states. For instance, analyzing HSP60 in conjunction with Iba1, CD11b, and TREM2 can clarify not only whether microglia are activated but also the pathways through which this activation occurs. The combined use of multiple markers, including HSP60, can improve diagnostic sensitivity and specificity, allowing for the detection of diverse activation states that might be missed when relying on a single marker. Through such comparative analysis, HSP60 has the potential to offer a more accurate evaluation of microglial activation when used alone or in combination with other markers. This represents a significant advancement for future research and clinical diagnostics.

Our study demonstrates that the expression of HSP60 is significantly upregulated on the surface of microglia activated by oxysterols such as 25OHChol and 27OHChol. These results suggest that HSP60 can serve as a specific marker for microglial activation and may play a role in modulating neuroinflammatory immune responses in the brain. This research provides insights into the mechanisms of microglial activation and contributes to the development of novel markers and treatments for neuroinflammatory conditions.

## 4. Materials and Methods

### 4.1. Cell Culture and Reagents

HMC3, a human microglial cell line, was purchased from the American Type Culture Collection (ATCC, Manassas, VA, USA). The cells were maintained in Dulbecco’s modified Eagle’s medium (DMEM) containing 10% fetal bovine serum (FBS) in the presence of penicillin and streptomycin. Cholesterol was purchased from Sigma-Aldrich (St. Louis, MO, USA), and 24sOHChol, 25OHChol, and 27OHChol were purchased from Santa Cruz Biotechnology (Santa Cruz, CA, USA). The lipids were dissolved in absolute ethanol and used as a stock concentrate of 2 mg/mL. The human β-amyloid peptide (1–42) (Aβ_1–42_) was purchased from Abcam (Cambridge, UK). Primary and secondary antibodies used in this study were purchased from Santa Cruz Biotechnology. Secondary antibodies, conjugated Alexa488 for immunofluorescence, were purchased from Invitrogen corporation (Waltham, MA, USA).

### 4.2. Isolation of Cell Surface Proteins

Proteins on the cell surface were isolated with the Pierce^®^ Cell Surface Protein Isolation Kit according to the manufacturer’s instructions (Pierce Biotechnology, Rockford, IL, USA). The brief procedure was carried out as detailed in the previous study [23].

### 4.3. Western Blot Analysis

HMC3 cells were lysed using Pro-Prep^TM^ lysis buffer (Intron Biotechnology, Sungnam, Korea). Proteins harvested from the cells were separated by SDS-PAGE and transferred onto a nitrocellulose membrane. After blocking the non-specific binding of the primary antibody with 5% skim milk in 0.1% Tween 20/TBS, the membrane was incubated with indicated primary antibodies at 4 °C overnight. The membrane was washed 3 times with 0.1% Tween 20/TBS for 15 min and was incubated with HRP-conjugated secondary antibodies for 1 h at room temperature. After washing 3 times with washing buffer for 15 min, the specific signals were detected with a PIERCE^®^ enhanced chemiluminescence Western blotting detection system (Pierce Biotechnology, Gainesville, FL, USA). Chemiluminescent images were captured using an Amersham Imager 600 (GE Healthcare Life Sciences, Pittsburgh, PA, USA).

### 4.4. Silver Staining

The isolated cell surface proteins were loaded on 10% SDS-PAGE gel, and the gel was stained with the PIERCE^®^ Silver Stain Kit (Pierce Biotechnology, Gainesville, FL, USA). This experiment was conducted in accordance with the user manual provided with the kit [23].

### 4.5. Matrix-Assisted Laser Desorption/Ionization Time-of-Flight (Maldi-Tof) Mass Spectrometry (MS)

Isolated protein band from the gel was analyzed with MALDI-TOF MS analysis, performed to obtain the spectrum of [M + H]^+^ molecular ions of the trypsinized fragments of the protein bands, as previously described in the study [24].

### 4.6. Immunocytofluorescence

The HMC3 cells were cultured on gelatin-coated coverslips (0.2% gelatin in PBS) and stimulated with Aβ_1–42_, cholesterol, or oxysterols for 48 h. The cells were fixed with 1% paraformaldehyde for 20 min and incubated with a blocking solution of 5% skim milk in PBS for 1 h. The cells were incubated at room temperature for 2 h with a primary antibody against heat-shock protein 60 (HSP60), which was diluted 1:100 in the blocking solution. The cells were washed 2 times with PBS for 5 min and were incubated at room temperature in the dark for 1 h with fluorescence-conjugated secondary antibodies, which were diluted 1:200 in PBS. Following another wash with PBS, the samples were mounted and visualized using a confocal microscope (FV1000; Olympus Corp., Tokyo, Japan).

## Data Availability

All data generated or analyzed during this study are included in this published article.

## Supplementary Materials

The following supporting information can be downloaded at: https://www.mdpi.com/article/10.3390/ijms25169073/s1.
